# Banana plant counting and morphological parameters measurement based on terrestrial laser scanning

**DOI:** 10.1186/s13007-022-00894-y

**Published:** 2022-05-18

**Authors:** Yanlong Miao, Liuyang Wang, Cheng Peng, Han Li, Xiuhua Li, Man Zhang

**Affiliations:** 1 Key Lab of Smart Agriculture System Integration, Ministry of Education, China Agricultural University, Beijing, 100083 China; 2 Key Lab of Agricultural Information Acquisition Technology, Ministry of Agriculture and Rural Affairs, China Agricultural University, Beijing, 100083 China; 3 College of Electrical Engineering, Guangxi University, Nanning, 530004 Guangxi China

**Keywords:** Banana, Counting, Pseudo-stem diameter, Pseudo-stem height, Terrestrial laser scanning, Point cloud

## Abstract

**Background:**

The number of banana plants is closely related to banana yield. The diameter and height of the pseudo-stem are important morphological parameters of banana plants, which can reflect the growth status and vitality. To address the problems of high labor intensity and subjectivity in traditional measurement methods, a fast measurement method for banana plant count, pseudo-stem diameter, and height based on terrestrial laser scanning (TLS) was proposed.

**Results:**

First, during the nutritional growth period of banana, three-dimensional (3D) point cloud data of two measured fields were obtained by TLS. Second, the point cloud data was preprocessed. And the single plant segmentation of the canopy closed banana plant point cloud was realized furtherly. Finally, the number of banana plants was obtained by counting the number of pseudo-stems, and the diameter of pseudo-stems was measured using a cylindrical segmentation algorithm. A sliding window recognition method was proposed to determine the junction position between leaves and pseudo-stems, and the height of the pseudo-stems was measured. Compared with the measured value of artificial point cloud, when counting the number of banana plants, the precision,recall and percentage error of field 1 were 93.51%, 94.02%, and 0.54% respectively; the precision,recall and percentage error of field 2 were 96.34%, 92.00%, and 4.5% respectively; In the measurement of pseudo-stem diameter and height of banana, the root mean square error (RMSE) of pseudo-stem diameter and height of banana plant in field 1 were 0.38 cm and 0.2014 m respectively, and the mean absolute percentage error (MAPE) were 1.30% and 5.11% respectively; the RMSE of pseudo-stem diameter and height of banana plant in field 2 were 0.39 cm and 0.2788 m respectively, and the MAPE were 1.04% and 9.40% respectively.

**Conclusion:**

The results show that the method proposed in this paper is suitable for the field measurement of banana count, pseudo-stem diameter, and height and can provide a fast field measurement method for banana plantation management.

## Background

Banana is the main cash crop in tropical and subtropical regions. The banana industry is an important pillar for rural economic prosperity and farmers’ poverty alleviation in many areas of southern China [1, 2]. The physical parameters of banana plant number, pseudo-stem diameter, and height are important morphological and phenotypic parameters of banana plants, and are closely related to the growth and yield of banana [3, 4]. Therefore, the measurement of morphological and phenotypic parameters is of great significance for banana production and field management. Manual measurement is subjective and inefficient; therefore, it is widely used to measure plant morphological and phenotypic parameters by image processing and 3D reconstruction [5–11].

Karami et al. [12] used an unmanned aerial vehicle (UAV) to obtain images and realize automatic counting of maize plants based on a genetic algorithm. Jiang et al. [13] realized rice ear counting based on a rice ear detection method to generate a feature pyramid. Based on crop images, crop plant counting can be achieved by improving deep learning methods. However, it is also easily affected by some factors, such as lighting conditions and imaging heights, and requires a large amount of data for model training [14, 15], which is not versatile among different crops. Wang et al. [16, 17] used binocular stereo vision to measure adult banana pseudo-stem diameter and height. The MAPEs of pseudo-stem diameter and height were 2.0% and 3.0%, respectively. However, single-plant acquisition did not achieve large-scale rapid measurements. Che et al. [18] used a camera to obtain images of crop stems under different illumination conditions and extracted stem edges. Zhang et al. [19] analyzed color image data, generated digital surface models and high-definition digital orthophoto images, and extracted field maize plant height, which could not realize single-plant measurements, and the measurement accuracy was low. Based on crop images, the plant height and stem thickness of crop plants can be measured by methods such as color information and texture features, however, they are sensitive to light and easily affected by background complexity [20, 21], and the measurement accuracy can be improved. Liang et al. [22] used continuously captured maize images and a motion restoration structure algorithm to realize 3D reconstruction of maize plants, and realized accurate and nondestructive measurements of 11 traits, such as plant height, stem diameter, and leaf area of corn. However, the measurement method of 3D reconstruction by image still has problems such as being easily affected by light and complicated algorithm. Based on image information, the parameters including crop counting, stem diameter, and stem height can be measured. However, there are still have some problems, such as difficulty in image segmentation, influence of illumination, lack of 3D information, requiring massive data calibration [23–25], improving the accuracy of measurement, and so on.

The point cloud data of crops obtained by 3D reconstruction can be used to extract high-precision morphological and phenotypic parameters [26, 27]. Song et al. [28] used Kinect V2 to obtain the diameter parameters of the banana pseudo-stem, selected the B-spline curve approximation method to fit the circumference of the banana pseudo-stem, used the particle swarm optimization algorithm to obtain the circle parameters based on the least squares method, and obtained a series of required parameters such as diameter and fitting circle center coordinates. The MAPE was 2.34%. Wang et al. [29] used statistical filtering for large-scale noise in the banana pseudo-stem point cloud and bilateral filtering to smooth small-scale noise. The pseudo-stem diameter and height of the banana were extracted from the processed point cloud, in which the MAPEs of the pseudo-stem diameter and height were 1.34% and 6.32%, respectively. Qiu et al. [30] used a Kinect camera to obtain the color and depth images of maize during the V9 period, obtained the stem trunk through image processing, and fitted the point cloud data to obtain the long and short axes of the maize stem. Based on the Kinect V2 point cloud data, the crop stem diameter and stem height can be measured. However, the data quality is easily affected by illumination and background complexity, and the measurement range is small. LiDAR has the advantages of little influence by light, high measurement accuracy, large measurement range, and 3D information. Zhang et al. [31] used airborne LiDAR to obtain forest point cloud data and used a tree height estimation method combining the canopy height model (CHM) and digital surface model (DSM) to measure forest height. Chatzinikos et al. [32] measured the heights of sunflowers, soybeans, and winter wheat using Vehicle LiDAR. Cai et al. [33] used backpack laser scanning (BLS) to obtain forest point cloud data, an irregular triangulation algorithm to remove ground points, and measured the DBH of trees through circle fitting. Huang [34] used BLS to collect forest point cloud data and achieved high-precision acquisition of tree DBH and height parameters. Based on the point cloud data of airborne LiDAR, vehicle LiDAR and BLS, the crop stem thickness and height can be measured, however, the measurement accuracy still needs to be improved. The UAV platform has the advantages of large measurement range and high efficiency. However, the information below the canopy is seriously lacking. The TLS point cloud data has the advantages of higher accuracy and higher density, and can obtain rich information below the canopy. Monika et al. [35] used TLS to obtain forest point cloud data, and measured the diameter at breast height (DBH), tree height and other parameters of trees. Cabo et al. [36] used handheld LiDAR and TLS to obtain forest point cloud data, and compared the accuracy of DBH extraction. Ma et al. [37] used TLS forest point cloud data and improved the k-means clustering method to extract the DBH of trees. Kankare [38] used TLS to obtain single-tree point cloud data, extract single-tree height, crown width, and other parameters, and established a single-tree biomass model. Tilly et al. [39] used TLS to obtain maize point cloud data, generate a crop surface model, and measure maize plant height. Su et al. [40] used TLS to obtain point cloud data of the maize VT period and extract the plant height. The parameters of plant stem diameter and plant height can be extracted using TLS. However, there is still the problem that the point cloud is missing due to crop occlusion, and it is difficult to separate the point cloud of a single plant from the population. At present, few studies have measured banana phenotypic parameters.

There were the following problems in using TLS to count banana plants and measure pseudo-stem diameter and height: bamboo poles supporting banana plants and broken leaves would interfere with banana plant counting; how to segment single banana plants from banana populations due to closed canopy and serious cross shielding of leaves; and the measurement of pseudo-stem height needs to identify the junction between the lowest leaf and pseudo-stem and the junction between the ground and pseudo-stem.

To solve the above problems, TLS was used to collect banana point cloud data in the nutrition growth period, and banana plant counting, pseudo-stem diameter, and height measurements were carried out. The number of plants was extracted from the fixed height point cloud data, and then a single plant was segmented using a combination of Euclidean clustering and K-means clustering. Finally, a method for calculating the pseudo-stem diameter and height of the banana pseudo-stem was proposed.

## Materials and methods

### Overview

**Fig. 1:**
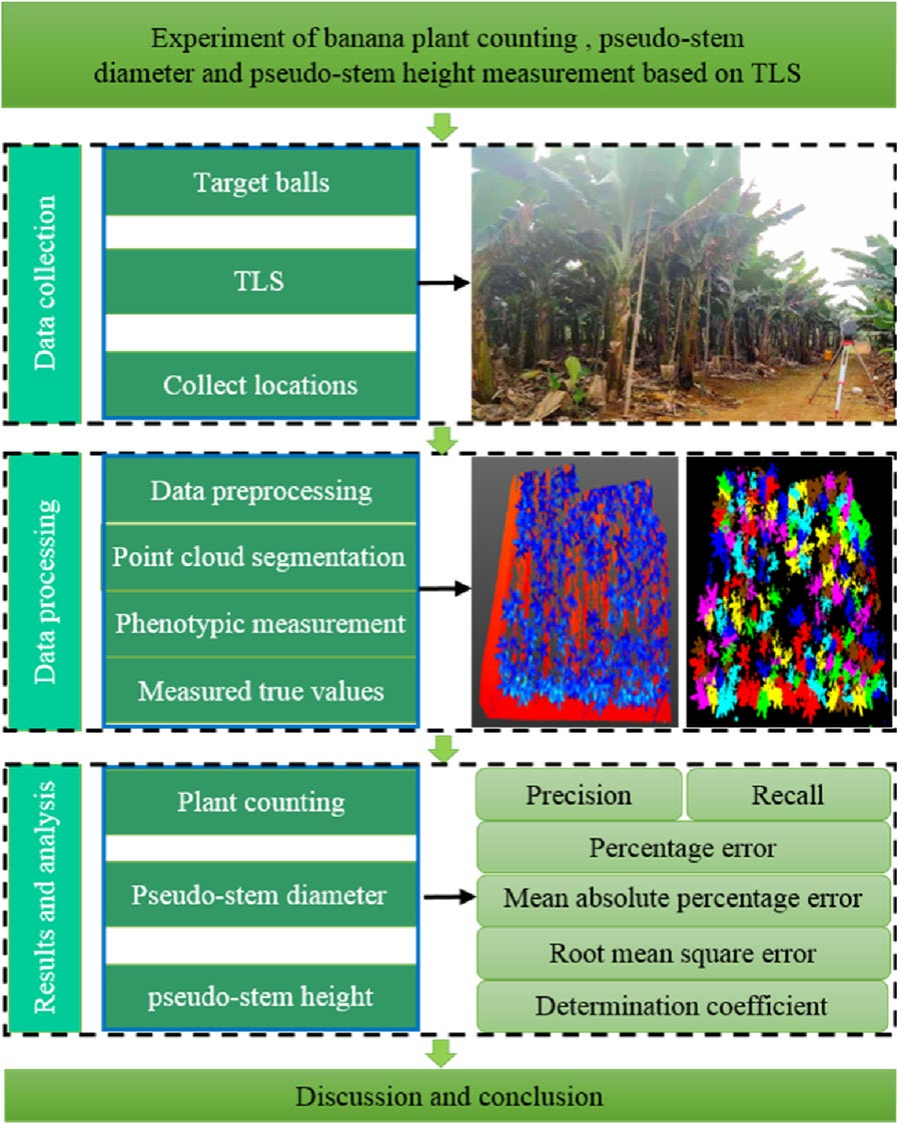
System overview

The overview of the system is illustrated in Fig. 1. To perform banana plant counting and pseudo-stem diameter and height measurements, banana plants were used as the research object for phenotypic parameter measurement experiments. During the experiments, the banana plants in the nutrition growth period were scanned using Trimble tx8 TLS to obtain the 3D point cloud data. Rapid measurements of banana plant count, pseudo-stem diameter, and height were realized using the point cloud processing method. Based on the point cloud data, the number of banana plants was counted manually, and the pseudo-stem diameter and height of the banana were measured as the true values. The banana phenotypic measurement algorithm was evaluated quantitatively by comparing the automatic measurement results with true values.

### Data collection

The data collection experiment dates were April 14 and April 17, 2021. The study area is part of the crop planting base of Guangxi Jiejiarun Technology Co., Ltd. (GJTCL), which is an area of approximately 140 ha located in Fusui County, Chongzuo City, Guangxi Zhuang Autonomous Region, China. This area lies between the lat. 22°29′34.58″N and lat. 22°30′48.32″N and long. 107°46′7.03″E and long. 107°47′19.70″E. The banana cultivar was Williams B6, and its growth process can be roughly divided into four phenological periods: sucker, nutritional growth, bud bursting, and fruit maturation periods. During the experiment, bananas were in the late stage of the nutritional growth period, and the canopy was closed.

**Fig. 2:**
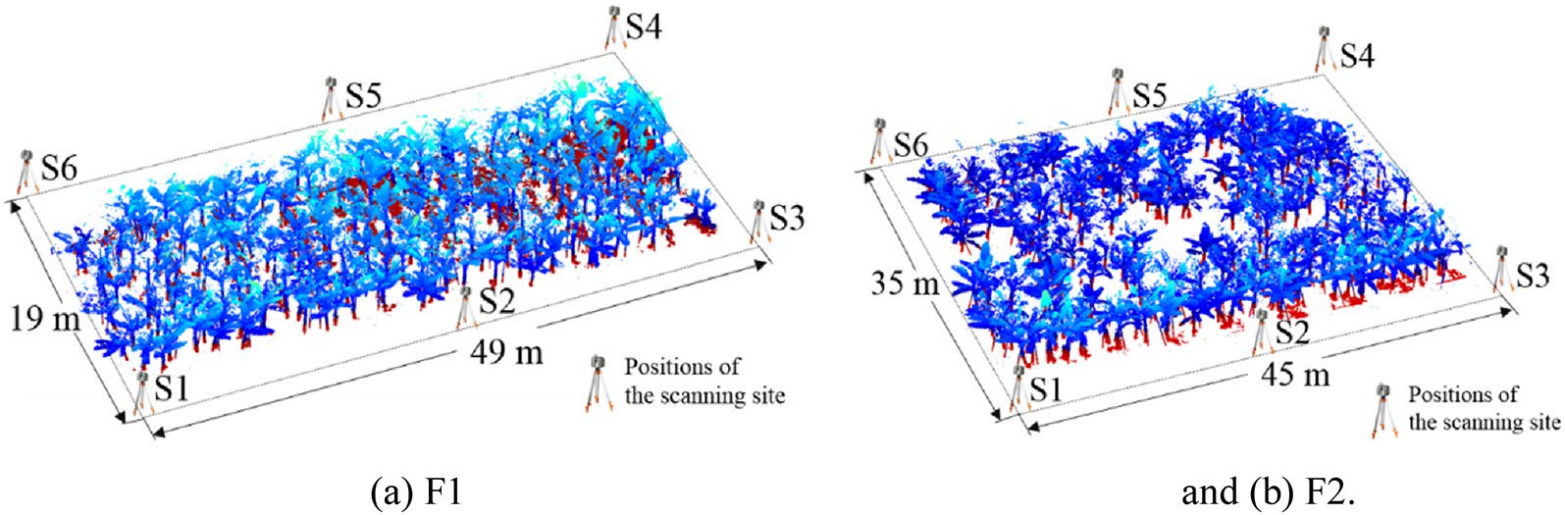
Positions of the scanning site on the two fields. S1, S2, S3, S4, S5 and S6 are the scanning stations 1 to 6 respectively. **a** F1 and **b** F2

The Trimble tx8 TLS of the American Trimble company was selected to collect 3D point cloud data during the banana nutrition growth period; its performance parameters are listed in Table 1. Two fields of banana gardens were scanned using the Trimble TX8. Field 1 (F1) was 49 m long and 19 m wide, with a field area of 931 m^2^, and field 2 (F2) was 45 m long and 35 m wide, with a field area of 1575 m^2^. Prior to the experiment, the target balls were placed in the collection point cloud data area. Each site cloud data and other site cloud data were required to include at least three common target balls, and the target balls were not collinear. Trimble tx8 was installed at the base of the tripod. The scanning density level was set to level 2 and the scanning time was 3 min. The three leveling screws under the equipment were adjusted to ensure that the equipment was perpendicular to the horizontal plane. The scan key was clicked to start the station scan. The scanning stations were evenly distributed according to the scanning area, and point cloud data of the six stations were obtained in each field. The total scanning time was 18 min for each field, including the time to move the TLS, the total time to acquire the point cloud per field was about 30 min. The positions of the scanning site on the two Fields are shown in Fig. 2. S1, S2, S3, S4, S5 and S6 are the scanning stations 1 to 6 respectively. To avoid missing the points describing the inner banana plant pseudo-stem, the distance between the scanning site positions should not be too far, and the banana plant should not be blocked between the TLS and the internal banana plant.

**Table 1:** Performance parameters of Trimble tx8 LiDAR

Specifications	Parameters	Specifications	Parameters
Field of view	360° × 317°	Scan distance	0.6–120 m
Laser class	Class 1—eye safe	Laser scan resolution	16″
Scan speed	1 MHz	Scanning accuracy	2 mm
Scan density	Level1/2/3, extended	Laser wavelength	1.5 μm
Data storage	USB3.0	Power	72 W
Operating temperature	0 ~ 40 ℃	Storage temperature	− 20 ~ 50 ℃

### 3D point cloud data processing

**Fig. 3:**
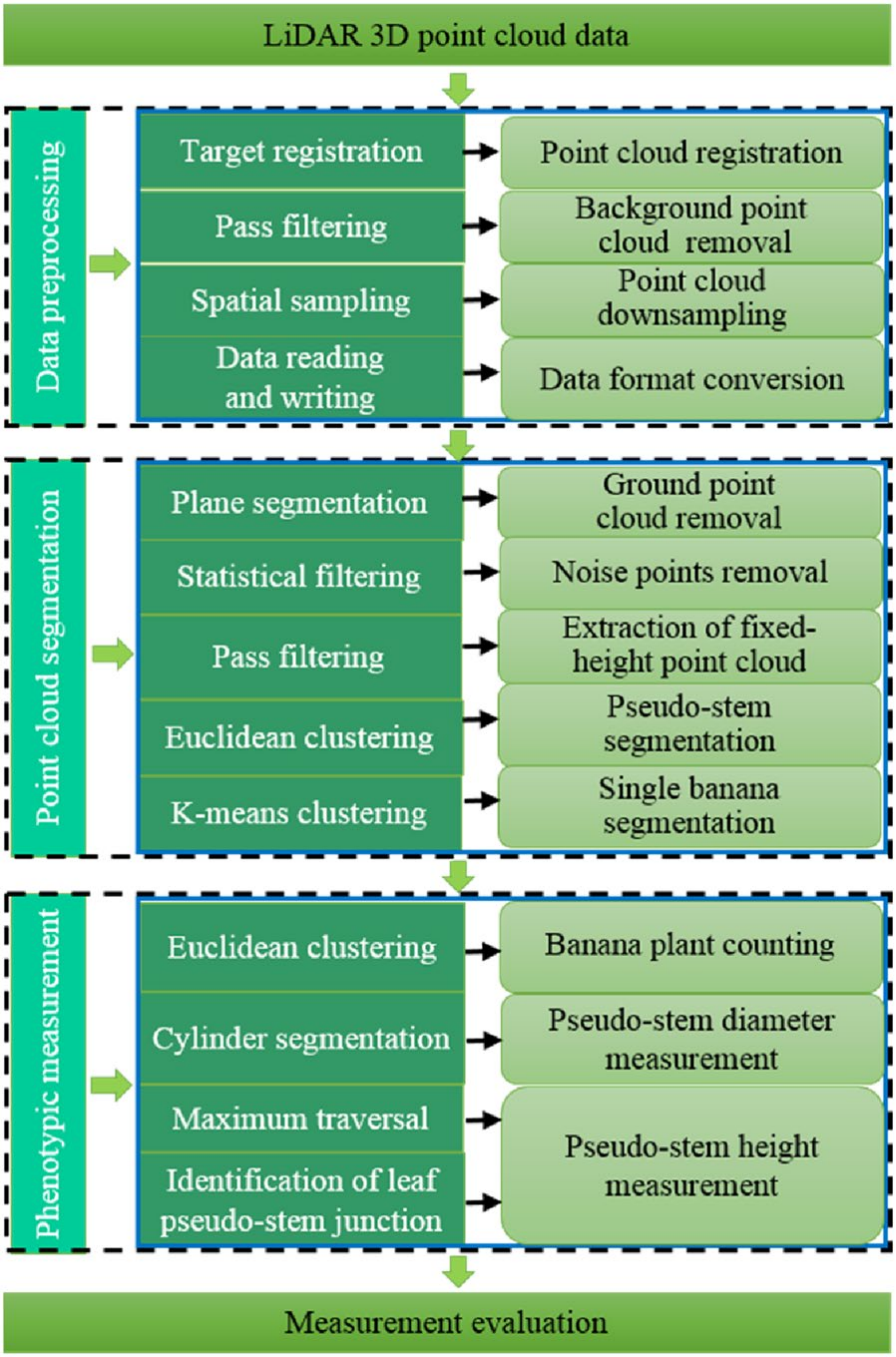
The overall point cloud data processing block diagram

The Trimble Realworks software was used as a 3D point cloud data processing tool to extract and match the point cloud data. With Visual Studio 2013 as the platform, Point Cloud Library 1.8.0 (PCL1.8.0) and Cmake3.8.0, were installed. To measure the number of banana plants, pseudo-stem diameter, and height based on the point cloud data in the field, C +  + was used for software programming. The processing of 3D point cloud data includes three parts: data preprocessing, point cloud segmentation, and banana phenotypic parameter measurement. An overall point-cloud data-processing block diagram is shown in Fig. 3.

### Data preprocessing

**Fig. 4:**
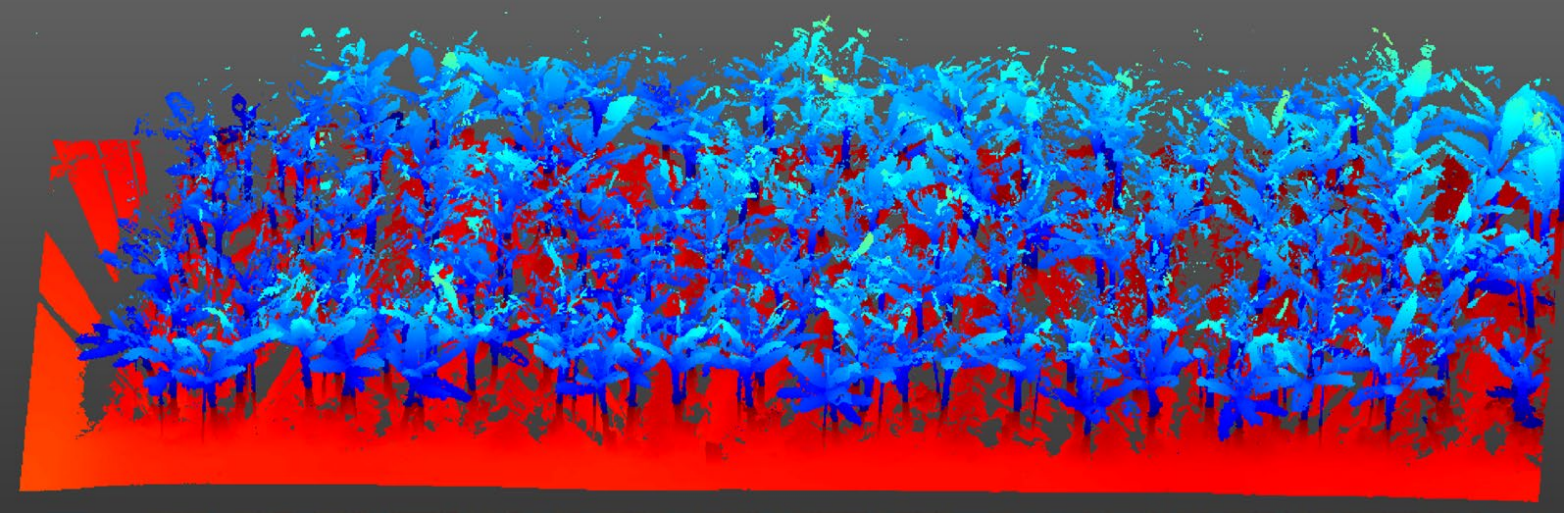
F1 point cloud

**Fig. 5:**
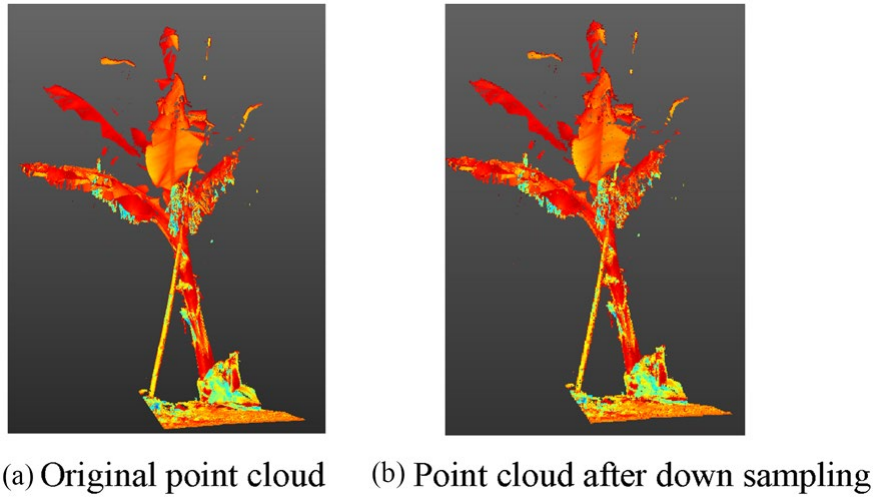
Down sampling. **a** Original point cloud. **b** Point cloud after down sampling

The data preprocessing procedure includes the following steps: point cloud data registration, background point cloud data removal, data downsampling, and format conversion. The banana point cloud data obtained from the field experiment were read using the Trimble Realworks software. The survey site cloud was established, and the point cloud data were registered using the target ball registration method. Through pass filtering, the background point cloud data were removed and the point cloud data of banana in the experimental area was extracted, F1, as shown in Fig. 4. The downsampling method was used for spatial sampling of the point cloud data in the experimental area, and the sampling distance was set to 8 mm. Compared with the original point cloud data, the number of point clouds was reduced from 100 million to 10 million. After downsampling, the outer contour of a single banana plant hardly changed; however, this did not affect the extraction of later character parameters, as shown in Fig. 5. The number of original point cloud is 761488, and the number of point cloud after down sampling is 124680. The point cloud data were saved in *.las format. To improve the efficiency of data processing and adapt to PCL applications, software was designed to convert the format of three-dimensional point cloud data from *.las to *.pcd.

### Banana point cloud segmentation

**Fig. 6:**
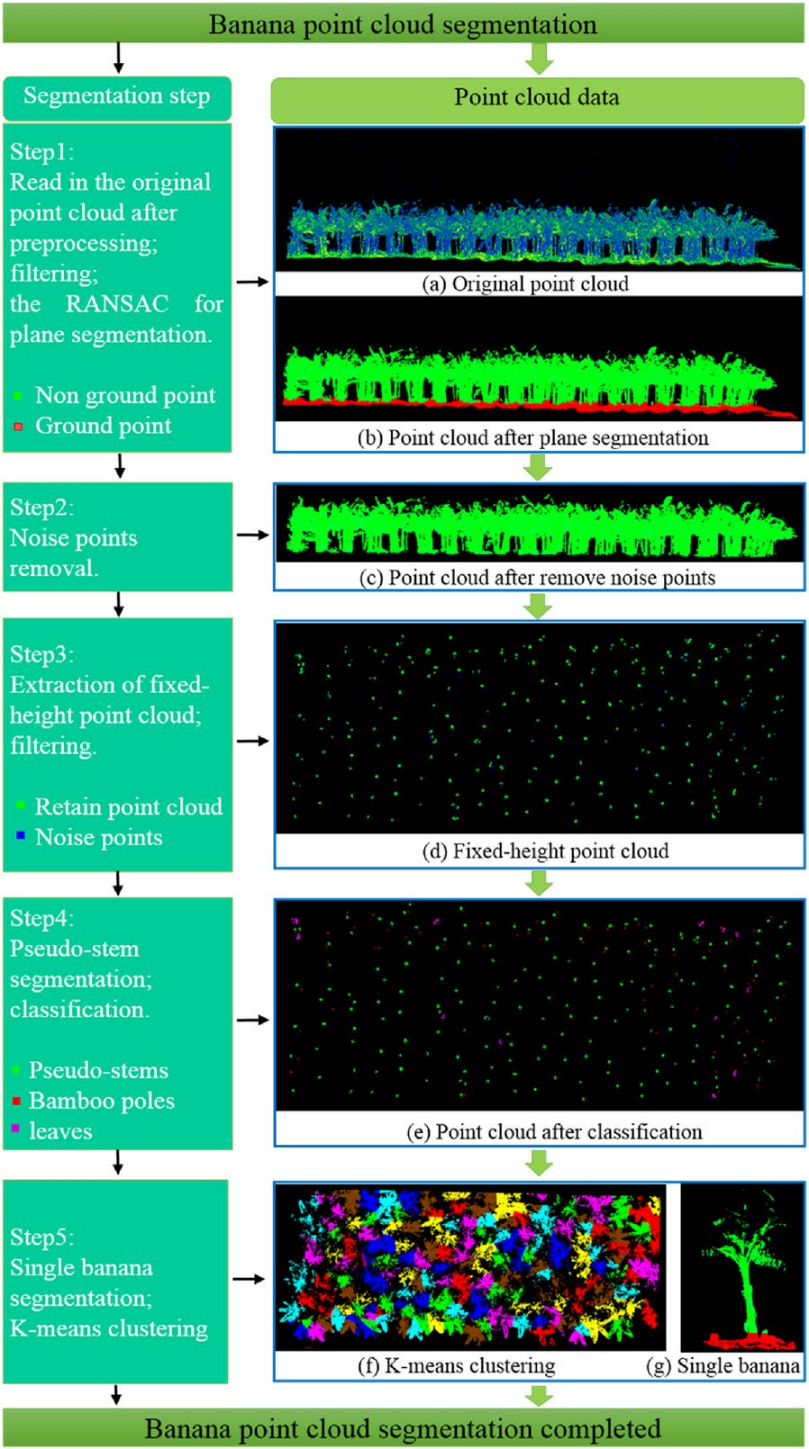
Banana point cloud segmentation. **a** Original point cloud; **b** Point cloud after plane segmentation; **c** Point cloud after remove noise points; **d** Fixed-height point cloud; **e** Point cloud after classification; **f** K-means clustering; **g** Single banana

Banana point cloud segmentation includes five steps: segmentation of banana plants and ground point cloud data, removal of outliers, extraction of fixed-height point cloud, pseudo-stem segmentation, and single plant segmentation. Taking F1 as an example, a point cloud segmentation process was introduced. The processing process is illustrated in Fig. 6. The pseudo code of the method is presented in Method. 1 (shown in Table 2). The specific processing steps are as follows:

**Table 2:**
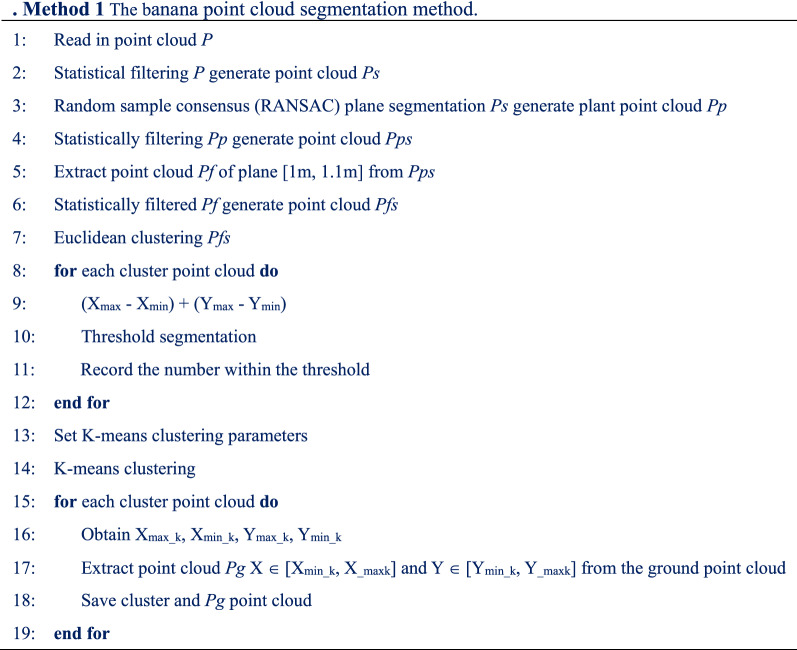
Method 1 The banana point cloud segmentation method

Step 1: The preprocessed original point cloud is illustrated in Fig. 6(a). First, to remove noise points, the point cloud data were statistically filtered. Second, the point cloud data were segmented using the random sample consensus (RANSAC) plane segmentation algorithm. The segmentation result is shown in Fig. 6(b), where the non-ground point cloud is displayed in green, the ground point cloud is displayed in red, and outliers in the air are removed.
Step 2: The segmented plant point cloud data were statistically filtered to remove noise points in the banana plants. The results after removal are shown in Fig. 6(c).
Step 3: The point cloud data within [1 m, 1.1 m] from the plane were extracted. For better Euclidean clustering, the extracted fixed-height point cloud data were statistically filtered to remove noise points around the pseudo-stem. The result is shown in Fig. 6(d). The reserved point cloud is displayed in green and the outlier point cloud is displayed in blue.
Step 4: The reserved point cloud was Euclidean clustering. The distances in the X-and Y-axes of each cluster of point cloud and their sum were calculated. The threshold was set to segment the pseudo-stem of a single banana plant from bamboo poles and broken leaves. The results are shown in Fig. 6(e). The banana pseudo-stems, broken leaves, and bamboo poles are shown in green, purple, and red, respectively.
Step 5: The number of segmented plant pseudo-stems was used as the K value in K-means clustering. The center point of the fixed-height pseudo-stem point cloud was used as the initialization center point for K-means clustering. The point clouds of the banana plants were clustered. The segmentation results are shown in Fig. 6(f). Different plant point clouds are displayed in different colors. For each type of plant point cloud, the range in the X-and Y-axes was obtained, and the ground point cloud data within the range were extracted. The point cloud of a single banana plant and the ground point cloud are shown in Fig. 6(g). The banana plant is displayed in green and the ground is shown in red.

### Measurement of banana phenotyping parameters

Based on the segmented fixed-height pseudo-stem and single-plant point cloud, the following methods were used to automatically measure the number of banana plants, pseudo-stem diameter, and pseudo-stem height within the field: euclidean clustering, random sampling consistency cylinder segmentation, maximum traversal, and leaf pseudo-stem junction recognition. The specific measurement methods for the phenotyping parameters were as follows:

#### Banana plant counting

The number of banana plants was calculated by counting the number of banana pseudo-stems using Euclidean clustering.

**Fig. 7:**
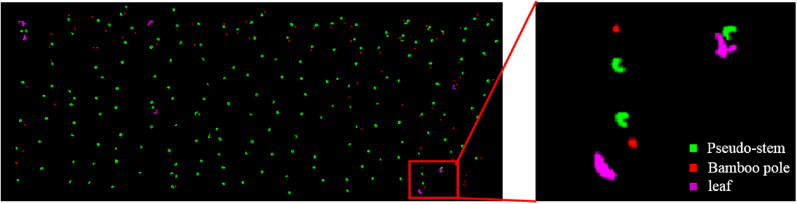
Bounding box threshold classification

To achieve high measurement accuracy, bamboo poles and leaves should be removed first. The distance sum in the X- and Y- axes of the bamboo pole point cloud was less than that of the banana pseudo-stem, and the distance sum in the X- and Y- axes of leaves point cloud greater than that of the banana pseudo-stem; therefore, the bamboo poles and leaves could be removed by setting the bounding box threshold, and the distance between the X- and Y- axes of the cluster point cloud was banana pseudo-stem within [0.25 m, 0.6 m]. The results are shown in Fig. 7. The banana pseudo-stems, bamboo poles, and broken leaves are shown in green, red, and purple, respectively.

#### Measurement of pseudo-stem diameter of banana

The measurement of the pseudo-stem diameter of a banana includes two parts: the extraction of a fixed height point cloud of a single banana and the measurement of the pseudo-stem diameter.

Fixed-height point cloud data of a single banana were extracted. The pseudo code of the method is presented in Method. 2 (shown in Table 3). The specific operations are listed below.

**Table 3:**
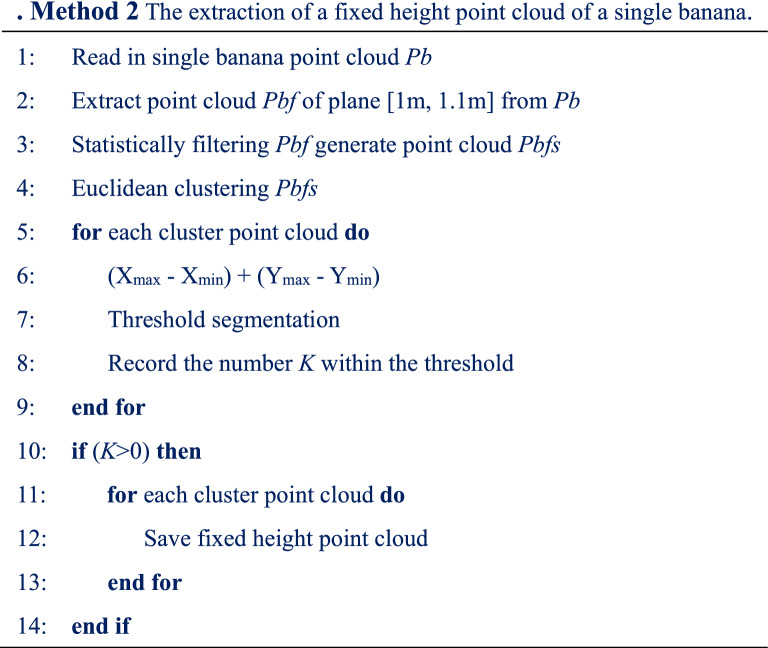
Method 2 The extraction of a fixed height point cloud of a single banana

Step 1: After K-means clustering, the single banana point cloud data are read in.
Step 2: The Pseudo-stem point cloud data within the fixed extraction distance plane [1 m, 1.1 m] were extracted.
Step 3: Statistical filtering was performed to remove noise points around the pseudo-stem.
Step 4: Euclidean clustering was performed on the filtered fixed-height point cloud data, and the number of threshold cohesion classes was counted, that is, the number of banana plants *K*.
Step 5: The number of banana plants, *K*, was determined. If the number of plants is *K* = 0, there is no operation; if the number of plants is *K* > 0, the *K* fixed-height pseudo-stem point cloud data is saved.
Step 6: All fixed height point cloud data for a single banana plant were extracted, and the program ended.

**Fig. 8:**
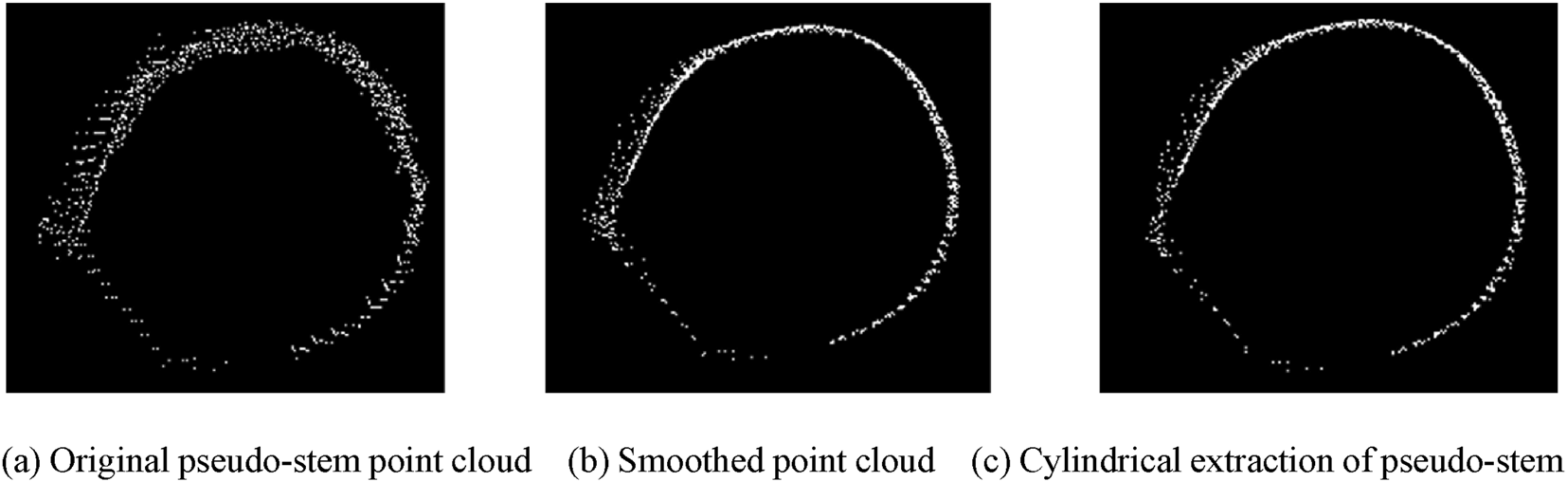
Pseudo-stem point cloud cylinder segmentation. **a** Original pseudo-stem point cloud. **b** Smoothed point cloud. **c** Cylindrical extraction of pseudo-stem

The extracted fixed-height point cloud data for a single banana are shown in Fig. 8(a). It could be seen that the thickness of the point cloud was large. For point cloud data, the moving least squares (MLS) algorithm was used to smooth the point cloud. The smoothing results are shown in Fig. 8(b). For the smoothed point cloud data, a random consistent cylinder was used to extract the point cloud. The results are shown in Fig. 8(c). The cylinder radius(r) was extracted, r multiplied by 2, that is, the pseudo-stem diameter of banana.

#### Measurement of pseudo-stem height of banana

The key to banana pseudo-stem height measurement is to identify the junction position between the banana leaf and pseudo-stem, and the junction position between the pseudo-stem and the ground. The distance between the two positions along the pseudo-stem direction was the pseudo-stem height. The junction position between the banana leaf and pseudo-stem is the boundary, and the banana plant is divided into two parts: the leaf canopy and the pseudo-stem. On the *XOY* plane, the perimeter of the circumscribed rectangle of the leaf canopy is longer than the perimeter of the circumscribed rectangle of the pseudo-stem. The perimeter of the circumscribed rectangle of the point cloud of different heights of the banana plant on the *XOY* plane is calculated, and the position of the sudden change in length is identified, that is, the junction position between the banana leaf and the pseudo-stem. The point cloud at the junction position between the pseudo-stem and the ground, which is located at the lowest point in the pseudo-stem point cloud. The point cloud data is obtained within the threshold of the distance axial vector of the pseudo-stem, and the lowest point is the junction between the pseudo-stem and the ground. The pseudo code of the method is presented in Method. 3 (shown in Table 4).

**Table 4:**
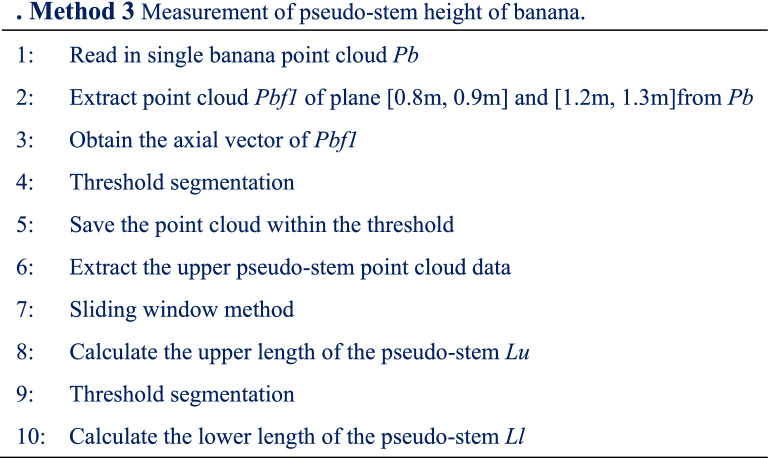
Method 3 Measurement of pseudo-stem height of banana

**Fig. 9:**
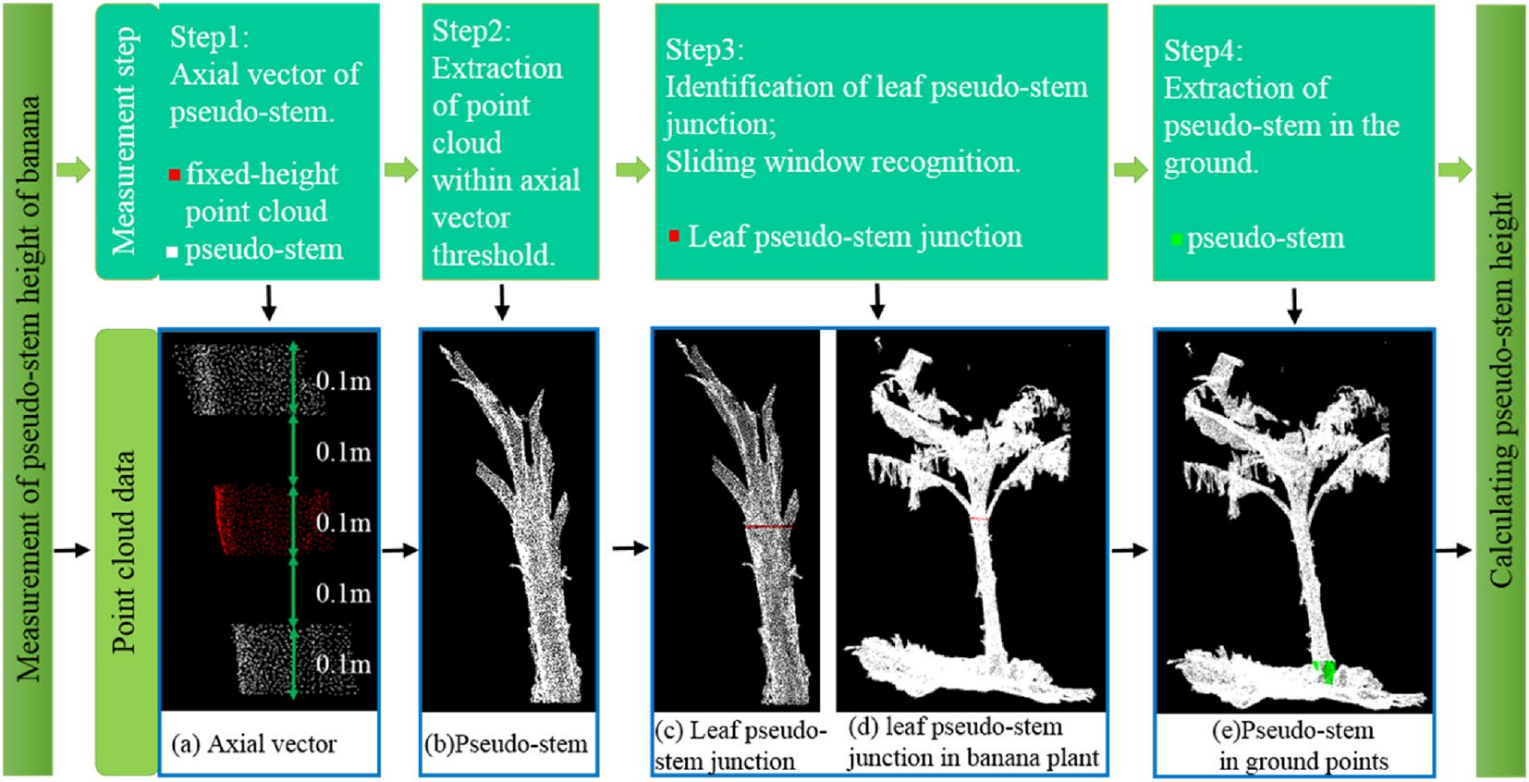
Measurement of pseudo-stem height of banana

Step 1: The axial vector of the banana pseudo-stem was obtained. Obtain the point cloud data of a fixed-height pseudo-stem point cloud 0.1 m upward and 0.1 m downward, as shown in Fig. 9(a). Point cloud smoothing and cylindrical segmentation were performed on the two point clouds of the pseudo-stem to obtain the axial vector of the banana and coordinates of a point on the axial vector.
Step 2: The distance from the point cloud of the banana plant to the axis vector was calculated. Based on the data processing experience, half of the pseudo-stem diameter of banana fixed height point cloud + 0.15 m was set as the threshold, the point cloud data, which less than the threshold was retained. The upper pseudo-stem point cloud data with Z coordinates greater than the minimum value of the Z coordinate of the fixed point cloud was extracted, as shown in Fig. 9(b).
Step 3: The sliding window was used to identify the junction position of the banana pseudo-stem and leaf. The pseudo code of the method is presented in Method. 4 (shown in Table 5).The sliding window recognition method includes the following steps:

1. Half of the pseudo-stem diameter r of banana fixed height point cloud and the upper pseudo-stem point cloud were read in, and the sliding distance d and width threshold w of the sliding window were set.
2. The lowest and highest points of the point cloud were obtained. Starting from the lowest point, the sum of x-axis distance a and y-axis distance b of the point cloud in each window was calculated. So r * 4 is approximately equal to *a* + *b*.
3. If *a* + *b* is greater than r * 4 + *w*, the window may contain leaves, and flag position is 1; otherwise, it is 0.
4. The longest continuous window sequence with the window flag position of 1 was counted, and the lowest number of sequences was obtained. Its height value was calculated as the return value of the function, and the pseudo stem point cloud with the height added to 3 * d was extracted as the input point cloud for the next execution. Because there were wilted petioles below the junction position between the banana leaf and pseudo-stem, the lowest number of sequences position may be below the wilted petiole. According to the principle of obtaining the lowest number of the longest sequence, in order to ensure that there was a point cloud of sufficient height above the junction position between the banana leaf and pseudo-stem, it was set to 3 * d.

**Table 5:**
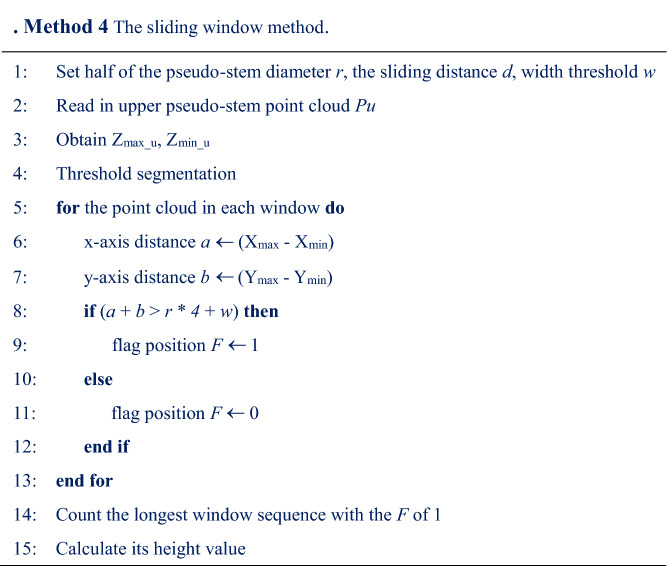
Method 4 The sliding window method

The exact position of the junction between the pseudo-stem and leaf was determined. As shown in Fig. 9(c), the red point cloud was used to identify the junction between the pseudo-stem and leaf. The identification position of the banana plant is shown in Fig. 9(d). It could be seen that the junction position of pseudo-stem and leaf could be accurately identified. The coordinates of the point with the largest z-axis on the pseudo-stem at the junction position were obtained by subtracting the coordinate value of the point on the axial vector to generate the upper vector, which was then multiplied by the axis vector point to calculate the upper length of the pseudo-stem.

Step 4: The ground point cloud data corresponding to each banana plant canopy were segmented from the overall ground point cloud, and then, the distance from the ground point cloud to the pseudo-stem axis vector was calculated. According to the data processing experience, half of the pseudo-stem diameter of banana fixed height point cloud + 0.15 m was set as the threshold, the point cloud data less than the threshold was retained, that is, the point cloud data of the pseudo-stem in the ground, as shown in Fig. 9(e), and the lowest part was the junction of the pseudo-stem and ground. The coordinates of the point with the smallest Z-axis on the pseudo stem point cloud were obtained by subtracting the coordinate value of the point on the axial vector to generate the lower vector. It was multiplied by the axis vector point to calculate the lower pseudo-stem length. The lower length was subtracted from the upper length and its absolute value was calculated. If it was greater than 5 m, the measurement failed; otherwise, it was the pseudo-stem height of the banana.

### Phenotyping parameters true values measurement

**Fig. 10:**
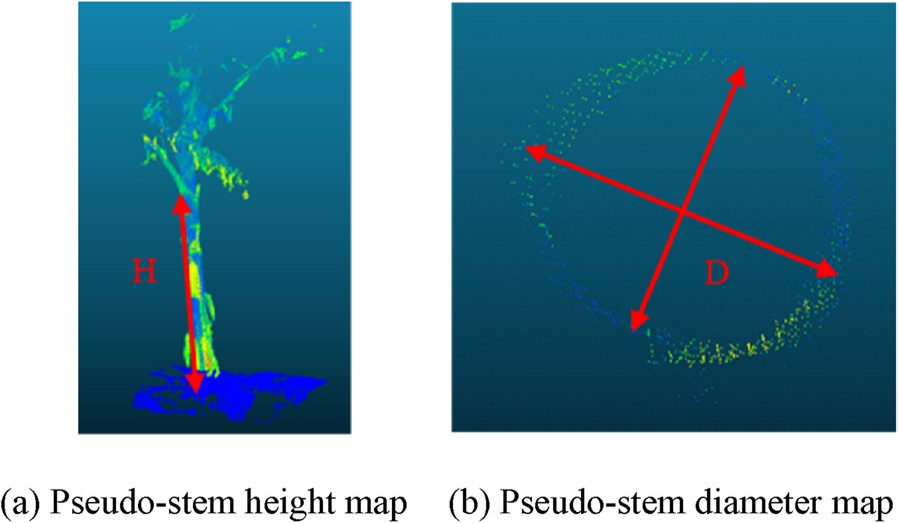
Measurement of pseudo-stem height and diameter of banana. **a** Pseudo-stem height map. **b** Pseudo-stem diameter map.

Trimble tx8 TLS can acquire millimeter-level point cloud data. Wind affects its measurement accuracy. The banana pseudo-stem will not change its shape due to the wind, which can ensure the accuracy of the point cloud collection. Studies have shown that artificial point cloud measurements can replace field measurements [41]. For the matched point cloud data, the number of banana plants was counted manually, and the pseudo-stem diameter and height of bananas were measured manually as the true value. A schematic of pseudo-stem height measurement is shown in Fig. 10(a). The distance from the junction between the lowest leaf and pseudo-stem of the banana plant to the junction between the ground on the same side and pseudo-stem is the pseudo-stem height H of the banana. The pseudo-stem point cloud data in the fixed height area were extracted, and a schematic diagram of the pseudo-stem diameter measurement is shown in Fig. 10(b). Because the banana pseudo-stem was similar to a cylinder, it was measured once at the maximum point cloud diameter and once at the minimum point cloud diameter, and the average value of the two measurements was calculated as the true value of the pseudo-stem diameter of the banana.

### Evaluation index

Precision (P), recall (R), and percentage error (PE) were used to quantify the performance of the banana plant counting algorithm. The accuracy of the designed method was evaluated by comparing the automatic measurement of false stem diameter and stem height with the measurement of the artificial point cloud. The measurement accuracy was evaluated by the mean absolute percentage error (MAPE), root mean square error (RMSE), and determination coefficient R^2^ (determination coefficient).

## Results and analysis

### Banana plant counting

**Fig. 11:**
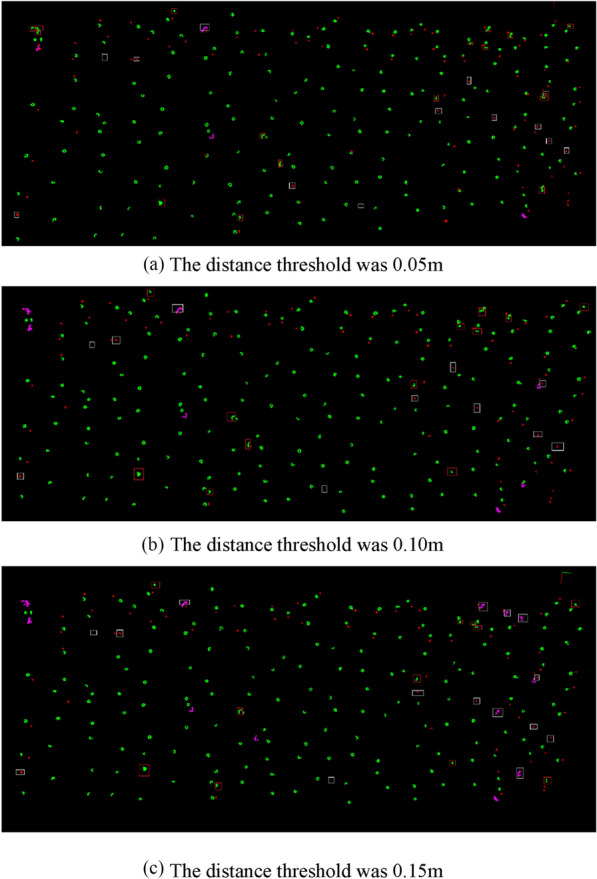
Threshold classification results. **a** The distance threshold was 0.05 m. **b** The distance threshold was 0.10 m. **c** The distance threshold was 0.15 m. Banana pseudo-stems are displayed in green, bamboo poles in red, and broken leaves in purple. The white box indicates that the plant was mistakenly identified as a bamboo pole or broken leaf, whereas the red box indicates that the bamboo pole or broken leaf was mistakenly identified as a plant

The distance threshold of Euclidean clustering has an important impact on the classification of banana pseudo-stems, bamboo poles, and leaves. If the threshold is set too small, a leaf will be divided into multiple classes, resulting in a distance between the X- and Y- axes and within the threshold range of the pseudo-stem. The point cloud at the edge of the pseudo-stem is filtered out, resulting in a smaller distance and in the direction of the X-and Y-axes, which will be misidentified as a bamboo pole. If the threshold value is set too large, the leaves, pseudo-stems, bamboo poles, and pseudo-stems will be divided into one class, resulting in an increase in the distance between the X- and Y- axes, which will be misidentified as leaves. When the distance threshold is set too large or too small, the counting error of banana plant number will increase; therefore, it is necessary to set an appropriate distance threshold. The distance thresholds were set as 0.05 m, 0.10 m and 0.15 m respectively. The recognition results of the banana plant algorithm are shown in Fig. 11, where the banana pseudo-stems, bamboo poles, and broken leaves are displayed in green, red, and purple, respectively. The white box indicates that the plant was mistakenly identified as a bamboo pole or broken leaf, whereas the red box indicates that the bamboo pole or broken leaf was mistakenly identified as a plant.

Under different distance threshold settings, the number of banana plants recognized by the algorithm and the number correctly recognized by the algorithm were counted. The calculation and evaluation indices used to measure the accuracy of the banana counting algorithm are shown in Table 6. Table 6 shows that with the increase in distance threshold, the number of algorithm recognitions decreased, mainly because plants and adjacent leaves or bamboo poles were recognized as one class, and the number of recognitions decreased. The correct number of algorithm recognitions increased first and then decreased, and reached the maximum when the distance threshold was set to 0.10 m. This was mainly because when the distance threshold was set to 0.05 m, a leaf was divided into multiple types of point clouds and misidentified as a pseudo-stem. The point cloud spacing at the edge of the pseudo-stem was large; therefore, it could not be classified as a pseudo-stem, which was misidentified as a bamboo pole. These reasons led to a low correct number when the distance threshold was set to 0.05 m. When the distance threshold was set to 0.15 m, the plant and adjacent leaves or bamboo poles were classified as a type of point cloud, which was incorrectly identified as leaves, resulting in a low correct number. Based on the evaluation index, the precision increased with an increase in the distance threshold. The recall was the highest when the distance threshold was set to 0.10 m; the percentage error was minimum when the distance threshold was set to 0.05 m or 0.10 m. When the distance threshold was set to 0.05 m, the precision and recall of the algorithm were low. When the distance threshold was set to 0.15 m, the recall of the algorithm was low and the percentage error was large. When the distance threshold was set to 0.10 m, the recall of the algorithm was high, the precision was close to the highest value, and the percentage error was small. By comprehensively comparing various evaluation indexes, the distance threshold was set to 0.10 m to count banana plants, and the number of plants was used as the K value in K-means clustering.

**Table 6:** Counting results of banana plants with different distance thresholds

Field	True banana number	Distance threshold/m	Algorithm recognition	Correct number	Precision	Recall	Percentage error
1	184	0.05	187	170	90.91%	92.39%	1.63%
		0.10	185	173	93.51%	94.02%	0.54%
		0.15	180	169	93.89%	91.85%	2.17%
2	200	0.05	194	182	93.81%	91.00%	3.00%
		0.10	191	184	96.34%	92.00%	4.50%
		0.15	187	182	97.32%	91.00%	6.50%

### Accuracy evaluation of pseudo-stem diameter and height of banana

The pseudo-stem diameter of the point cloud after K-means classification was measured. A total of 187 banana plants were detected in F1, and 175 banana diameters were measured by cylindrical segmentation, of which only two were mistakenly identified as plants and 11 banana plants were not detected. In F2, 192 bananas were detected, and 177 banana diameters were measured by cylindrical segmentation, of which only one was mistakenly identified as a plant by the leaf. A total of 176 plants were correctly measured, and 24 banana plants were not detected. The main reason why the existing plants were not detected was that point cloud occlusion led to the loss of a fixed-height pseudo-stem point cloud, resulting in a smaller bounding box. Alternatively, the pseudo-stem was close to the broken leaf, resulting in the enlargement of the bounding box beyond its threshold range of the bounding box.

**Fig.12:**
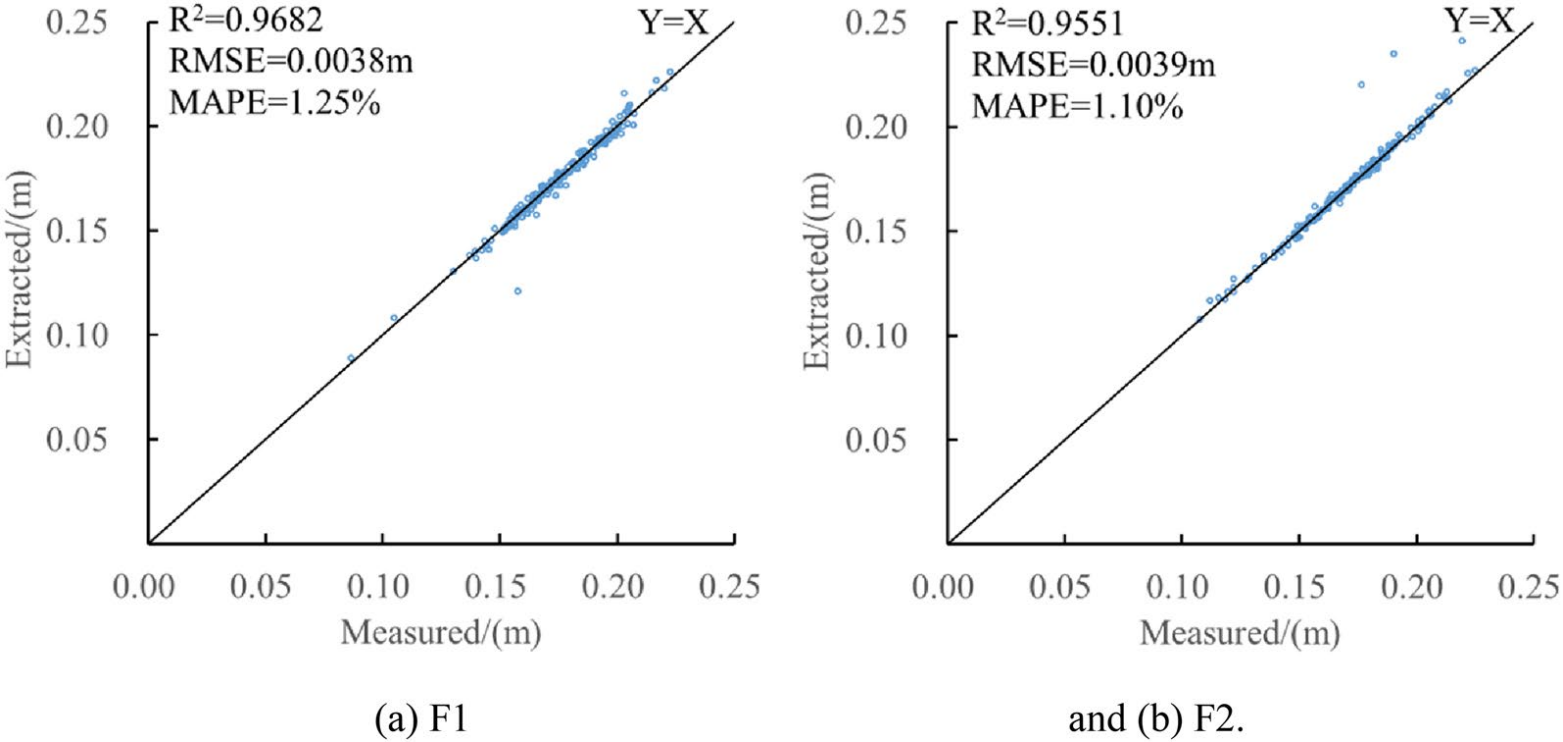
Comparison between measured and extracted of pseudo-stem diameter. **a** F1 and **b** F2

The banana pseudo-stem diameters of F1 and F2 were automatically and correctly measured using the program, and the results were compared with the artificial point cloud measurement results, as shown in Fig. 12. The banana pseudo-stem diameter was measured using automatic methods in different fields. The R^2^ was more than 0.95; the RMSE was controlled within 0.40 cm; the MAPE is not more than 1.30%, and the accuracy of automatic measurement of pseudo-stem diameter is not less than 98.70%. The results showed that this method had high accuracy in measuring the banana pseudo-stem diameter, and the measured value of the algorithm was consistent with the true value measured by the artificial point cloud.

After cylinder segmentation, the point cloud was recognized by the sliding window, the pseudo-stem height was measured, and the pseudo-stem heights of 175 bananas were measured in F1. Only two of these were mistakenly identified as plants by the leaves. In F2, the stem height measurement, only 1 of 177 bananas was mistakenly identified as plants by leaves, two pseudo-stems were measured for more than 5 m, and 174 plants were measured correctly. The pseudo-stem height measurement results exceeded the threshold mainly because point cloud occlusion led to the loss of banana leaf point clouds, and the contact position between the leaf and banana pseudo-stem could not be detected.

**Fig.13:**
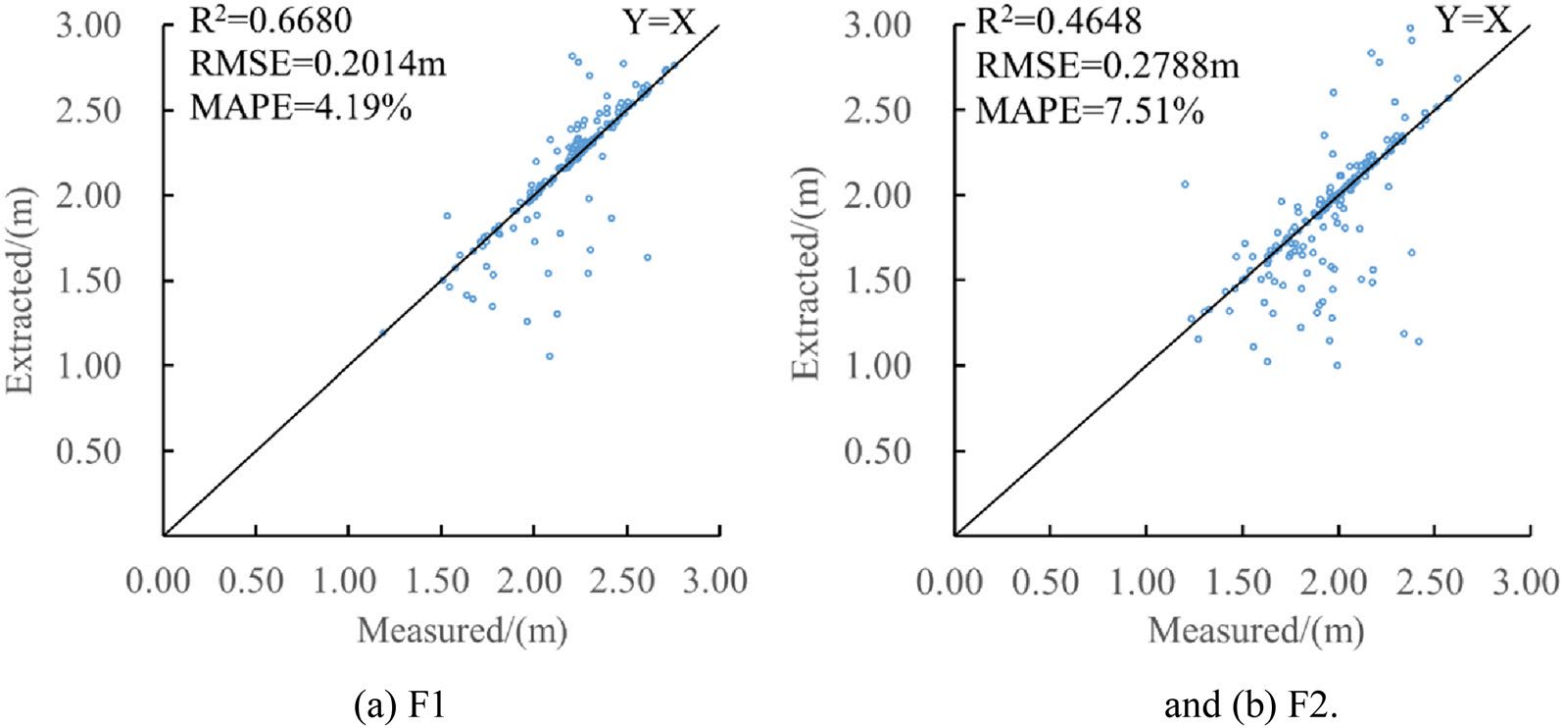
Comparison between measured and extracted of pseudo-stem height. **a** F1 and **b** F2

The banana pseudo-stem heights of F1 and F2 were automatically and correctly measured using the program, and the results were compared with the artificial point cloud measurement results, as shown in Fig. 13. Banana pseudo-stem height was measured using automatic measurement methods in different fields. The R^2^ was more than 0.46; the main reason was that the bamboo pole supported the banana plant, resulting in the low measured value of some pseudo-stem heights, and the banana pseudo-stem height was concentrated at 2.2 m, and the measurement error had a great impact on R^2^. The RMSE was controlled within 0.28 m; the MAPE was not greater than 7.60%, and the accuracy of program automatic measurement of pseudo-stem diameter was not less than 92.40%. The results showed that this method had a high accuracy in measuring banana pseudo-stem height, and the measured value of the algorithm was consistent with the true value measured by the artificial point cloud.

It can be seen from the automatic measurement results that the measurement accuracy of the pseudo-stem diameter is higher than that of the pseudo-stem height. The main reason is that, in the measurement of pseudo-stem diameter, threshold segmentation was used to extract fixed high pseudo-stem point cloud data, remove the interference of bamboo poles and leaves, and ensure the accuracy of cylindrical fitting. In the measurement of pseudo-stem height, there was no algorithm design for banana plants supported by bamboo poles, which leads to serious interference of bamboo poles on the measurement of pseudo-stem height of some banana plants. F1 covers an area of 931 m^2^ and F2 covers an area of 1575 m^2^. The phenotypic measurement results showed that F1 was better than F2 in terms of the measurement accuracy of plant number count, pseudo-stem diameter, and height. The main reason is that the area of F2 is larger than that of F1, and the density of the point cloud of F2 is lower than that of F1, which results in the missing of the petiole point cloud of some banana plants, and the measured value was too large. The point cloud is segmented into banana plants and bamboo poles, and the number of scanning stations is reasonably set, which can improve the accuracy of pseudo-stem height measurement.

## Discussion

The purpose of this study is to use TLS to collect high precision 3D point cloud data of banana plants in the fields, and to realize the automatic and accurate measurement of the number of banana plants, and pseudo-stem diameter and height.

Compared to manual measurement, six scan stations were arranged for each field. The time of one scan is 3 min, the total scanning time is 18 min, considering the time of moving the TLS, the total time to acquire the point cloud per field is about 30 min. It takes an average of 3 min to manually measure the pseudo-stem height and diameter of a banana plant. There are 184 banana plants in F1, and the total time is 552 min. There are 200 banana plants in F1, and the total time is 600 min. Manual measurement takes approximately 20 times than TLS.

Compared with TLS maize plant counting, Lin et al. [42] realized the automatic registration algorithm of point cloud based on the target ball, and the counting error was 7.9–13.9%. In this study, the counting error was 0.54–4.50%, and the counting error was reduced by 7.36–9.40%. However, we relied on Trimble Realworks software for point cloud data extraction and registration, which reduced the automation and efficiency of the banana phenotypic parameter measurement.

Compared with Kinect V2 banana pseudo-stem diameter and height measurement, The MAPEs for diameter measurement were 1.34% and 2.34% [28, 29]. In this study, the MAPEs were 1.10% and 1.25%, and the MAPE decreased by 0.24–1.09%. The MAPE for height measurement was 6.32% [29]. In this study, the MAPEs were 4.19% and 7.51%, and the MAPEs were basically the same. The method of this paper realized the data segmentation of a single banana plant point cloud, which effectively avoided the interference of bamboo poles and broken leaves on banana point cloud segmentation.

However, in pseudo-stem segmentation, the existing segmentation methods should be optimized to improve the segmentation accuracy; the method of segmenting bamboo and banana point clouds from point clouds should also be added to provide better point cloud data for subsequent phenotypic measurement. In the part of phenotypic parameter measurement, because there was a common value range between bamboo pole diameter and banana pseudo-stem diameter, there will be errors in banana plant counting using this method, which will also interfere with the measurement accuracy of pseudo-stem height. Therefore, it is necessary to further optimize the phenotypic parameter measurement methods. In addition, the density of the scanning station should be further optimized to achieve a higher measurement accuracy and efficiency.

In the next step, the bamboo pole segmentation algorithm in the point cloud processing process will be added, which can remove the bamboo pole point cloud through the morphological difference between the bamboo pole and the banana pseudo-stem, and improve the measurement accuracy of banana plant count and pseudo-stem height. In addition, other banana phenotypic parameters, such as leaf length, leaf width, and leaf area, can be measured using 3D point cloud data. In future research, it will be extended to the measurement of other phenotypic parameters to improve the utilization efficiency of point cloud data.

## Conclusions

- (1) Banana plants during the nutritional growth period were used as the research object. TLS was used to perform field-level banana plant number counting, banana single plant segmentation, and rapid measurement of pseudo-stem diameter and height.
- (2) By counting the K value in K-means clustering, the single plant segmentation of the canopy closed banana plant point cloud was realized, and the number of banana plants were counted automatically.
- (3) The cylinder segmentation method was used to measure the diameter of the banana pseudo-stem.
- (4) The sliding window along the axis of the banana pseudo-stem was used to identify the junction position between the banana pseudo-stem and the leaf. The junction position between the banana pseudo-stem and the ground was obtained, and the banana pseudo-stem height was measured.

## Data Availability

The datasets used and/or analyzed during the current study available from the corresponding author on reasonable request.
